# Posterior Reversible Encephalopathy Syndrome (PRES)

**DOI:** 10.21980/J85W6C

**Published:** 2020-01-15

**Authors:** William Ciozda, Adeola Adekunbi Kosoko

**Affiliations:** * McGovern Medical School at the University of Texas Health Science Center at Houston, Department of Emergency Medicine, Houston, TX

## Abstract

**Audience:**

Emergency medicine residents of all levels

**Introduction:**

Posterior reversible encephalopathy syndrome (PRES) is a clinically significant cause of seizures, headache, neurologic deficit, and hypertensive emergency that is not uncommon in the emergency department. Posterior reversible encephalopathy syndrome was initially described as a clinical syndrome in 1996.^1^ It is an important cause of hypertensive emergency that is not often covered in depth in the emergency medicine curriculum since the true incidence and disease process continues to be researched.

Populations who are at most risk for PRES include those with chronic hypertension, chronic renal disease, autoimmune disease, and immune suppression.^2^ Patients with PRES will often present with varied forms of encephalopathy and sometimes even focal neurologic symptoms that would suggest a cerebral vascular accident. These neurologic symptoms can include visual complaints and headache. Seizures are also frequently reported in association with PRES.^3^

Early identification and appropriate management of PRES decreases morbidity and mortality without chronic neurologic sequelae. The pillars of diagnosis and management can be initiated in the emergency department. This includes a diagnosis made by a thorough history and physical exam and cerebral imaging.^4^ The mainstay of management is parenteral anti-hypertensives with proper blood pressure monitoring.^5^

**Educational Objectives:**

By the end of the simulation, the learner will be able to: 1) manage an acute seizure 2) discuss imaging modalities to diagnose PRES 3) discuss medical management of PRES.

**Educational Methods:**

This simulation exercise is meant to be presented as a traditional medium-to-high-fidelity medical simulation case. With minor adjustments, it could be utilized as a low-fidelity case or an oral exam case.

**Research Methods:**

The educational content and general usefulness of this simulation was evaluated by open verbal (qualitative) feedback from a convenience sample of random participants following a completion of the case and debriefing by a participant group (n=30) of emergency medicine residents at a large 3-year residency training program.

**Results:**

The overall feedback was positive. Participants felt that it was a good opportunity to practice identifying PRES and managing it in a safe learning environment. They especially appreciated learning more about the pathophysiology of PRES, the high-risk factors for PRES, and management of the condition.

**Discussion:**

Posterior reversible encephalopathy syndrome, an uncommon condition, presents similar to many other benign and common complaints. It is crucial to be able to differentiate PRES from other causes of headache, visual disturbance, and seizures. It is important to keep PRES in mind when considering hypertensive emergencies. Many PGY-1 residents struggled to diagnose and treat PRES because it was often not on their differential, and this case helped broaden their differential. PGY-2 and PGY-3 were more frequently able to appropriately diagnose and treat PRES in this patient but found the case to be helpful in their decision-making and learning more about PRES pathophysiology. This case and associated high-yield debriefing session were effective for learners of all levels.

**Topics:**

Posterior reversible encephalopathy syndrome (PRES), altered mental status, seizure, headache, hypertensive emergency.

## USER GUIDE

**Table:** 

List of Resources:	
Abstract	46
User Guide	48
Instructor Materials	50
Operator Materials	62
Debriefing and Evaluation Pearls	65
Simulation Assessment	69

**Learner Audience:**

Interns, junior residents, senior residents

**Time Required for Implementation:**

Instructor Preparation: 20 minutes

Time for case: 15–20 minutes

Time for debriefing: 15–30 minutes

**Recommended Number of Learners per Instructor:** 3–4

**Topics:**

Posterior reversible encephalopathy syndrome (PRES), altered mental status, seizure, headache, hypertensive emergency.

**Objectives:**

By the end of the simulation, the learner will be able to:

1. Review the management of an acute seizure
2. Discuss imaging modalities to diagnose PRES
3. Discuss medical management of PRES and participants did not identify any major areas of change in the simulation scenario.

### Linked objectives and methods

PRES is a rare syndrome that has the potential for neurologic damage. It is becoming ever more recognized due to increased awareness and diagnostic modalities. PRES can have a wide range of clinical presentations but involves a syndrome of hypertension and seizures. It is important for the emergency medicine physician to be able to diagnose and appropriately treat PRES. This simulation will challenge the learners to obtain a thorough history and physical exam (objective 1) in order to develop a differential diagnosis for headache, altered mental status, and seizures (objective 2). As the learners pursue a workup for this patient, they should be able to manage the patient’s symptoms of a refractory headache (objective 3) and an acute seizure when the patient has a first-time seizure. PRES could mimic status migrainosus and can cause seizures and sometimes even status epilepticus. Imaging is necessary to diagnose PRES, especially because cerebrovascular accident (stroke) is on the differential diagnosis for the presenting symptoms of the patient. The learners will be able to discuss the different imaging modalities with their benefits and limitations. Once the diagnosis of PRES is made, the learners should be able to initiate appropriate medical management, including appropriate specialty consultation and disposition.

### Recommended pre-reading for instructor

- Neill, TA. Reversible posterior leukoencephalopathy syndrome. Post TW, ed. UpToDate. Waltham, MA: UpToDate Inc. Accessed on January 12, 2020. https://www.uptodate.com/contents/reversible-posterior-leukoencephalopathy-syndrome
- Hobson EV, Craven I, Blank SC. Posterior Reversible Encephalopathy Syndrome: A Truly Treatable Neurologic Illness. *Peritoneal Dialysis International*. 2012;32(6):590–594.

### Results and tips for successful implementation

This scenario was designed for residents and medical students to diagnose, appropriately treat, and disposition a patient with PRES in the emergency department. This case was designed to be performed using high-fidelity simulation but can also be performed using lower fidelity or as an oral exam case. The case allows learners to work through a broad differential for headache and altered mental status, ultimately treating and diagnosing PRES and seizures as an associated complication.

We conducted a pilot session of this simulation case with approximately 30 emergency medicine residents and medical students in groups of 4–6 which included MS4 medical students and PGY-1 to PGY-3 residents. The educational content and general usefulness of this simulation was evaluated by open verbal (qualitative) feedback from a convenience sample of random participants following a completion of the case and debriefing by a participant group (n=30) of emergency medicine residents at a large 3-year residency training program. Five resident participants and 2 medical students at random provided the most useful feedback for *this* simulation case rather than about simulation in general (Table 1).

The case was challenging to the more novice learners (4^th^ year medical students and PGY-1 residents) who often were unable to make the diagnosis of PRES. Many of them explained that they were not particularly familiar with the diagnosis. The PGY- 2 and PGY-3 residents found the case to be useful and were more likely to make the diagnosis and treat PRES appropriately, though many of them also had knowledge gaps identified by this simulation. They were glad for the opportunity to review the pathophysiology and preferred management for PRES. Due to the observations and feedback, we recommend that if interns are given this case, they should be placed in groups with senior residents who may be more familiar with PRES. Participant feedback was generally positive during post-session, and participants did not identify any major areas of change in the simulation scenario.

## Supplementary Information



## Figures and Tables

**Table:** 

#1	Point of care glucose
#2	Complete blood count
#3	Basic metabolic panel
#4	Coagulopathy panel
#5	Venous blood gas
#6	Erythrocyte sedimentation rate (ESR) and C-reactive protein
#7	Troponin-I
#8	Electrocardiogram (ECG)
#9	Chest Radiograph (CXR)
#10	Computed tomography (CT) head without contrast
#11	Magnetic resonance imaging (MRI) brain without contrast

**Table:** 

*Point of Care Glucose*	110 mg/dL

**Table:** 

**Vitals:**	*Heart Rate (HR)* 105	*Blood Pressure (BP)* 226/115	*Respiratory Rate (RR)* 18
	*Temperature (T)* 98.5°F axillary	*Oxygen Saturation (O_2_Sat)* 98% on room air	

**Table:** 

*Complete blood count (CBC)*	
White blood count (WBC)	6.8 x1000/mm^3^(H)
Hemoglobin (Hgb)	11.1 g/dL
Hematocrit (HCT)	39.0%
Platelet (Plt)	335 x1000/mm^3^

**Table:** 

*Basic metabolic panel (BMP)*	
Sodium	134 mEq/L
Chloride	99 mEq/L
Potassium	5.8 mEq/L
Bicarbonate (HCO_3_)	26 mEq/L (L)
Blood Urea Nitrogen (BUN)	8 mg/dL (H)
Creatine (Cr)	1.9 mg/dL (H)
Glucose	104 mg/dL

**Table:** 

*Coagulation Studies*	
International Normalized Ratio (INR)	1.14
Prothombin Time (PT)	27 seconds
Activated Partial Thromboplastin Time (aPTT)	32 seconds

**Table:** 

*Venous Blood Gas (VBG)*	
pH	7.35
pCO2	40 mmHg
pO2	35 mmHg
HCO3	27 mEq/L
Lactic Acid	1.3 mEq/L

**Table:** 

*C-Reactive Protein*	5 mg/L
*Erythrocyte Sedimentation Rate*	1 mm/Hg

**Table:** 

*Troponin-I*	*0.06 ng/mL*

**Figure:**
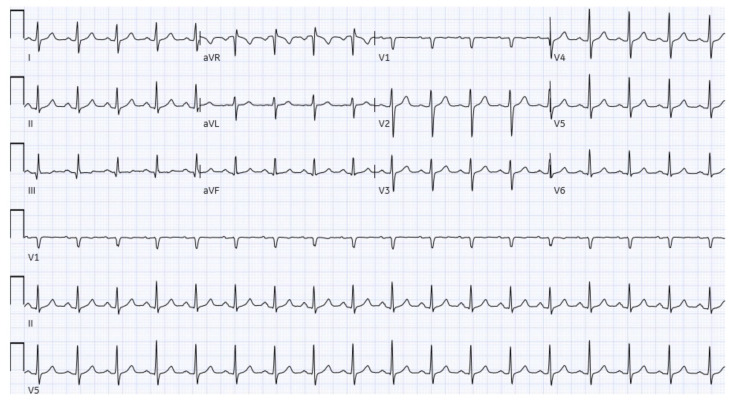


**Figure:**
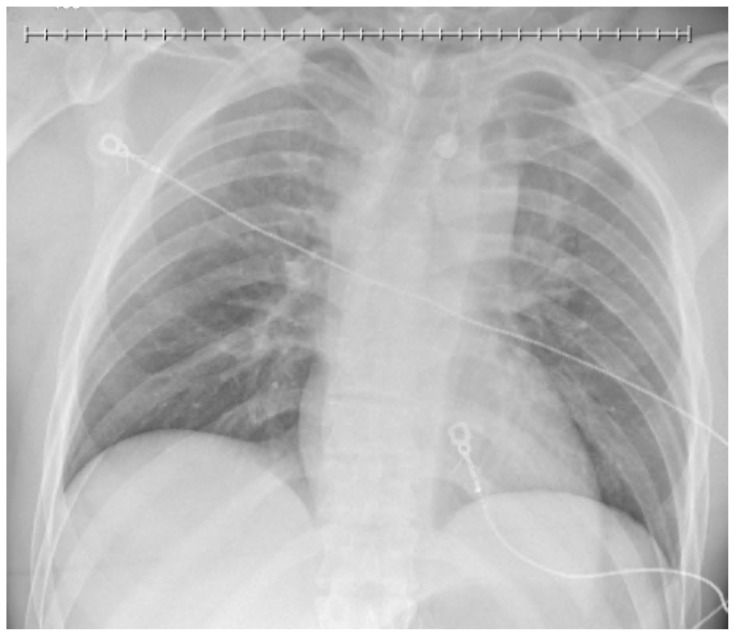


**Figure:**
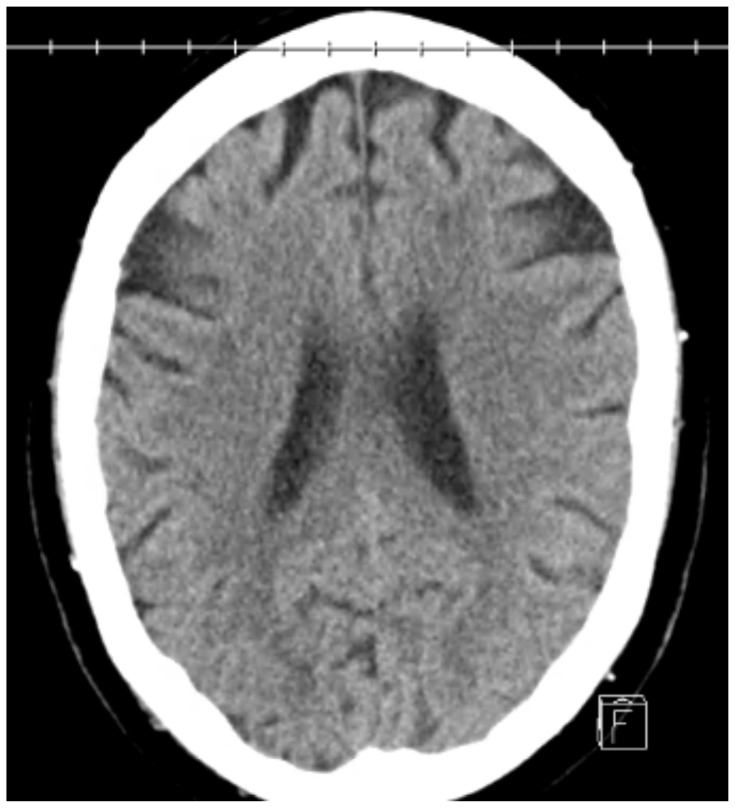


**Figure:**
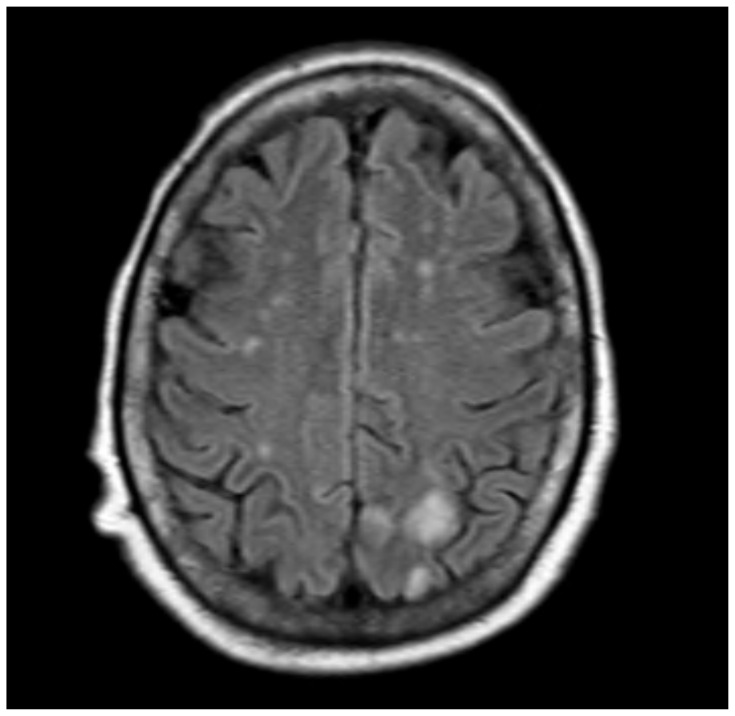


**Figure:**
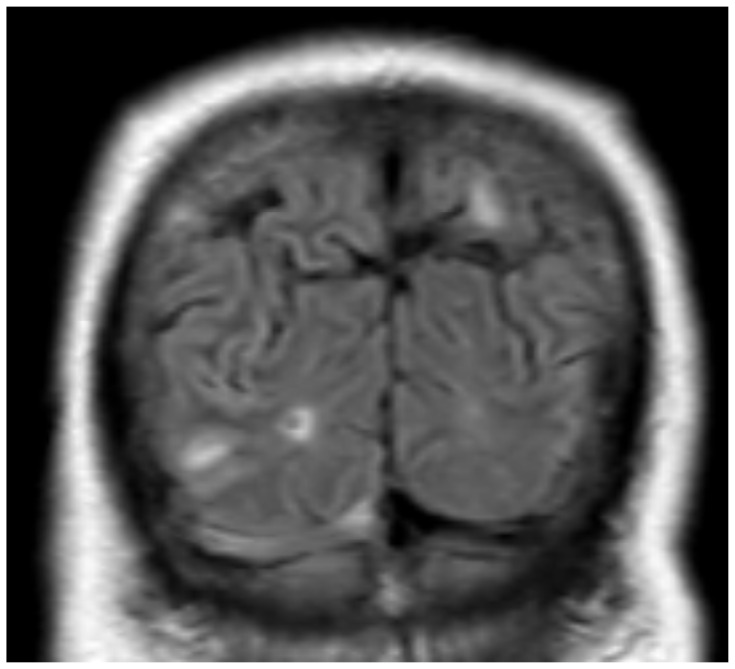


**Table:** SIMULATION EVENTS TABLE

Minute (state)	Participant action/ trigger	Patient status (simulator response) & operator prompts	Monitor display (vital signs)
0:00 (Baseline)	Introductions		T 98.5°F HR 105 BP 226/115 RR 18 O2 98%
	Attach the patient to cardiac monitor and pulse oximeter	Patient will have a chief complaint of headache and blurry vision	
	Obtain vital signs	Patient will be lying in bed in mild distress due to headache	
	Perform primary survey		

1:00	Obtain full history and physical exam	Patient will have a normal neurological exam with no acute neurological deficits	HR 105 BP 226/115 RR 18 O2 98%
	Obtain IV access		

03:00	Participants should treat the patient’s headache	If symptom treatment initiated: Blood pressure will improve, and patient will have only mild improvement in symptoms (pain score 5/10)	If headache treated: T 98.5°F HR 100 BP 216/101 RR 18 O2 98%
	Participants should initiate a work-up with lab tests and radiographic imaging	If symptom treatment is not initiated: Patient will complain of worsening pain (pain score 7/10) and nurse will ask participants if symptoms can be treated	
		If there is administration of antihypertensives, the blood pressure will improve, but the next step in the case is for the patient to have a seizure, regardless of whether this intervention is initiated	If headache not treated: T 98.5°F HR 110 BP 230/120 RR 19 O2 98%

06:00	Patient will be pending radiographic studies	While patient is awaiting results, patient will have a generalized tonic-clonic seizure	Following seizure: T 98.5°F HR 120 BP 240/115 RR 12 O2 90%
	Laboratory studies will slowly result		

08:00	Participants should recognize and manage the patient’s seizure	The patient should receive supplemental oxygen and be placed in the recovery position. The participants should time the seizure	If blood pressure untreated: T 98.5°F HR 105 BP 239/120 RR 17 O2 98%
		The patient will have a 1-minute seizure and will not require anti-epileptic medications.	
		Optional: For a more complex case, the patient may have status epilepticus (>5 minutes of seizures or repeated seizures without returning to baseline).	
		The patient would then require anti-epileptic medications either rectally, IV, or intramuscularly (IM)	

10:00	Following return of radiographic imaging, the participants should identify that this patient has PRES	If participants are unable to recognize the diagnosis, nursing staff will state “I’m concerned about that blood pressure. Can we give the patient something for that?”	If blood pressure untreated: T 98.5°F HR 105 BP 239/120 RR 17 O2 98%
	Learners should initiate IV antihypertensive medications	If the patient’s elevated blood pressure is not addressed, patient will have another seizure (patient will return to neurological baseline between seizures)	If blood pressure is treated: T 98.5°F HR 90 BP 204/105 RR 15 O2 98%

12:00	Participants should speak to neurology consult over the phone	If diagnosis is unclear, neurology consultant will ask for further work-up including CT scan or MRI	If blood pressure untreated: T 98.5°F HR 105 BP 239/120 RR 17 O2 98%
		If blood pressure is not controlled or not adequately controlled, neurology consultant will ask for blood pressure control with goal systolic blood pressure <170 mmHg	
		If learners do not consult neurology, the nurse should ask, “Is there anyone we can ask for help? This blood pressure seems dangerous.”	If blood pressure is treated: T 98.5°F HR 90 BP 168/89 RR 15 O2 98%

13:00 Case Completion	Participants should admit the patient to the intensive care unit (ICU)	The patient should be admitted to the intensive care unit for continued management	

**Figure:**
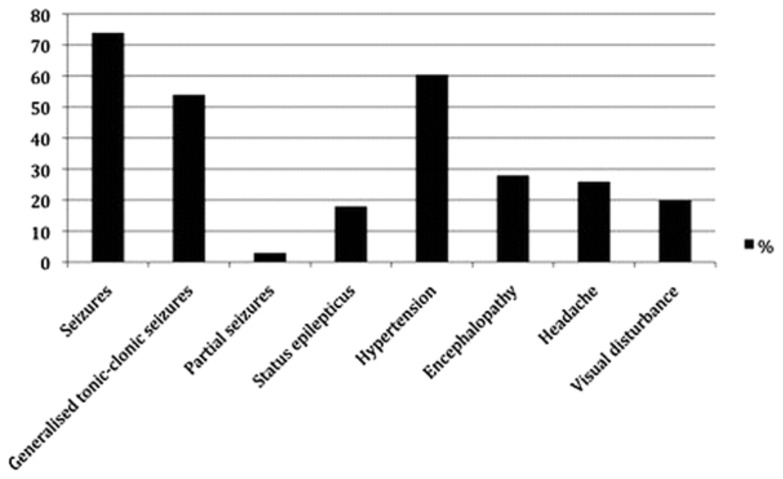
Frequency of posterior reversible encephalopathy syndrome signs and symptoms seen in a series of 120 cases (Fugate, et. al, 2010)

**Table:** 

**Critical Actions** Connect the patient to the cardiac monitor and obtain a full set of initial vital signs Obtain IV access and laboratory studies Symptomatically treat the patient’s headache – antiemetics, analgesics (avoiding opioids), antihistamines, vasodilators, abortive agents, etc. Manage a patient with a seizure – including supplemental oxygen, recovery position, possibly benzodiazepines or other antiepileptic agents Obtain appropriate head/brain imaging Make the diagnosis of posterior reversible encephalopathy syndrome (PRES) Begin appropriate blood pressure management with IV antihypertensives Obtain a neurology consultation Admit the patient to the intensive care unit (ICU)	0:00

**Table:** 

	Milestone	Did not achieve level 1	Level 1	Level 2	Level 3
1	**Emergency Stabilization (PC1)**	□ Did not achieve Level 1	□ Recognizes abnormal vital signs	□ Recognizes an unstable patient, requiring intervention Performs primary assessment Discerns data to formulate a diagnostic impression/plan	□ Manages and prioritizes critical actions in a critically ill patient Reassesses after implementing a stabilizing intervention
2	**Performance of focused history and physical (PC2)**	□ Did not achieve Level 1	□ Performs a reliable, comprehensive history and physical exam	□ Performs and communicates a focused history and physical exam based on chief complaint and urgent issues	□ Prioritizes essential components of history and physical exam given dynamic circumstances
3	**Diagnostic studies (PC3)**	□ Did not achieve Level 1	□ Determines the necessity of diagnostic studies	□ Orders appropriate diagnostic studies. Performs appropriate bedside diagnostic studies/procedures	□ Prioritizes essential testing Interprets results of diagnostic studies Reviews risks, benefits, contraindications, and alternatives to a diagnostic study or procedure
4	**Diagnosis (PC4)**	□ Did not achieve Level 1	□ Considers a list of potential diagnoses	□ Considers an appropriate list of potential diagnosis May or may not make correct diagnosis	□ Makes the appropriate diagnosis Considers other potential diagnoses, avoiding premature closure
5	**Pharmacotherapy (PC5)**	□ Did not achieve Level 1	□ Asks patient for drug allergies	□ Selects an medication for therapeutic intervention, consider potential adverse effects	□ Selects the most appropriate medication and understands mechanism of action, effect, and potential side effects Considers and recognizes drug-drug interactions
6	**Observation and reassessment (PC6)**	□ Did not achieve Level 1	□ Reevaluates patient at least one time during case	□ Reevaluates patient after most therapeutic interventions	□ Consistently evaluates the effectiveness of therapies at appropriate intervals
7	**Disposition (PC7)**	□ Did not achieve Level 1	□ Appropriately selects whether to admit or discharge the patient	□ Appropriately selects whether to admit or discharge Involves the expertise of some of the appropriate specialists	□ Educates the patient appropriately about their disposition Assigns patient to an appropriate level of care(ICU/Tele/Floor) Involves expertise of all appropriate specialists
9	**General Approach to Procedures (PC9)**	□ Did not achieve Level 1	□ Identifies pertinent anatomy and physiology for a procedure Uses appropriate Universal Precautions	□ Obtains informed consent Knows indications, contraindications, anatomic landmarks, equipment, anesthetic and procedural technique, and potential complications for common ED procedures	□ Determines a back-up strategy if initial attempts are unsuccessful Correctly interprets results of diagnostic procedure
20	**Professional Values (PROF1)**	□ Did not achieve Level 1	□ Demonstrates caring, honest behavior	□ Exhibits compassion, respect, sensitivity and responsiveness	□ Develops alternative care plans when patients’ personal beliefs and decisions preclude standard care
22	**Patient centered communication (ICS1)**	□ Did not achieve level 1	□ Establishes rapport and demonstrates empathy to patient (and family) Listens effectively	□ Elicits patient’s reason for seeking health care	□ Manages patient expectations in a manner that minimizes potential for stress, conflict, and misunderstanding. Effectively communicates with vulnerable populations, (at risk patients and families)
23	**Team management (ICS2)**	□ Did not achieve level 1	□ Recognizes other members of the patient care team during case (nurse, techs)	□ Communicates pertinent information to other healthcare colleagues	□ Communicates a clear, succinct, and appropriate handoff with specialists and other colleagues Communicates effectively with ancillary staff

**Table 1:** Learner’s feedback responses to PRES simulation session (PGY, Post-Graduate Year).

Feedback	Level of Training
*“I thought it was good, because we don’t always get excited about high blood pressures that we see on the monitor regularly.”*	PGY-2
*“The simulation taught me to always keep PRES on my differential diagnosis.”*	PGY-2
*“I just need to learn about PRES, I don’t know much about it.”*	PGY-1
*“I have to go and read more at home.”*	PGY-2
*“We thought it was meningitis!”*	PGY-1
*“I’ve never heard of this.”*	PGY-1
*“It was helpful that our PGY-3 knew what was going on. I was confused.”*	PGY-1
*“I knew some of things about it, but I didn’t know all the features. This was a good synopsis. I liked the slides.”*	PGY-2
*“I had never heard of this before.”*	4^th^ year Medical Student
*“This was extremely helpful getting to review hypertensive emergencies.”*	PGY-3
*“I’ve heard people say the term, but I guess I didn’t really know about the diagnosis.”*	PGY-2

## INSTRUCTOR MATERIALS

**Case Title:** Posterior Reversible Encephalopathy Syndrome (PRES)

**Case Description & Diagnosis (short synopsis):** The patient is a 63-year-old female with a past medical history of hypertension and renal transplant four years prior. She presents to the emergency department (ED) with a complaint of a new headache. She will be hypertensive upon evaluation but not demonstrate any focal neurologic defects. The learner is expected to treat her for status migrainosus or another cause of refractory headache. However, her headache does not remit with medications. The patient has a non-sustained seizure and is briefly post-ictal. Because this is a first-time seizure, she should receive a CT scan of her head. The learner should recognize the patient’s history and hypertension, seizure, and headache clinically suggest posterior reversible encephalopathy syndrome (PRES) and should manage the patient with intravenous (IV) antihypertensives. She will require intensive care unit (ICU) admission and neurology specialist evaluation.

### Equipment or Props Needed

High-fidelity simulator/mannequin

Cardiac monitor

Pulse oximeter

Blood pressure cuff

Intravenous (IV) bags and tubing

Prop medications – IV Syringes, IV Bags

Nasal cannula

Non-rebreather oxygen mask

Bag valve mask

Crash cart with defibrillator

Intubation equipment (including endotracheal tube, stylet, 10cc syringe, and laryngoscope)

### Confederates needed

Roles can be played by a single operator or multiple persons as available.

- Nurse to assist with management of the patient
- Neurology specialist for consultation and ICU admission
- Critical care physician for ICU admission

### Stimulus Inventory

**Background and brief information:** The scenario takes place in a tertiary/quaternary emergency department. The patient is a 60-year-old woman with a complaint of an unremitting headache.

**Initial presentation:** The patient has come from home by private vehicle and has been triaged by nursing as an ESI (Emergency Severity Index) level 2. She is next in queue for medical evaluation. She is laying in the stretcher, alert, and in no acute distress.

### How the scenario unfolds

The patient is a 63-year-old woman who is laying in the bed in no acute distress. She has been experiencing a headache all morning. Learners should rapidly assess the patient’s airway, breathing, circulation, and for disability. She should be placed on full cardiopulmonary monitors and learners should obtain a full set of vital signs. The patient has stable airway, breathing, circulation, and does not demonstrate acute neurologic deficit. Vital signs are notable for mild tachycardia and severe hypertension. The learners should obtain a full history and cardiovascular- and neurologic-focused physical exam. The patient’s history is significant for a renal transplant and hypertension. Her physical exam is non-focal.

The learner is expected to treat her headache pain with headache abortive agents (eg, IV fluids, antihistamines, analgesics, anti-psychotics, magnesium sulfate, anti-emetics, etc.), keeping status migrainosus in their differential diagnosis. Learners should place an IV line and draw laboratory studies which they feel are appropriate. They may even give the patient oxygen, considering a cluster headache in the differential diagnosis.

Regardless of which medications the patient receives, the patient continues to complain of headache. She has a non-provoked, non-sustained general tonic-clonic seizure. Afterwards, she is post-ictal for a brief period. She will not require airway assistance or even antiepileptics, though some learners may be aggressive in prescribing these interventions.

This is the first time the patient has ever had a seizure, so learners are expected to pursue emergency brain imaging, which will likely be a computed tomography (CT) scan of the head.

The CT scan will not be concerning for mass lesion, acute intracranial bleed, or ischemic stroke. The learner should recognize the patient’s history and hypertension, seizure, and headache clinically suggest posterior reversible encephalopathy syndrome (PRES). They should manage the patient with intravenous antihypertensives (eg, labetalol, nicardipine or nitroprusside) and consider magnetic resonance imaging (MRI) of the brain. The patient’s headache will improve with normalized blood pressure measurements. The learner should also consult a neurologic specialist and a critical care physician for an intensive care unit (ICU) admission. Because the patient is having a hypertensive emergency, she should receive an arterial line for adequate blood pressure monitoring.

If the patient does not have appropriate blood pressure management, she will have another general tonic-clonic seizure.

If the MRI brain is obtained, it will show radiographic findings consistent with PRES.

Optional: For senior learners or for improperly managed PRES, the patient may have status epilepticus at the time of her new-onset seizure: a seizure lasting >5 minutes or 2 or more seizures in a 5-minute period without returning to baseline. She would require benzodiazepines and possibly fosphenytoin/phenytoin, valproic acid, propofol, levetiracetam or another antiepileptic drug. She would also require airway assistance with bag valve mask and/or intubation.

### Critical Actions

1. Connect the patient to the cardiac monitor and obtain a full set of initial vital signs
2. Obtain IV access and laboratory studies
3. Symptomatically treat the patient’s headache – antiemetics, analgesics (avoiding opioids), antihistamines, vasodilators, abortive agents, etc.
4. Manage a patient with a seizure – including supplemental oxygen, recovery position, possibly benzodiazepines or other antiepileptic agents
5. Obtain appropriate head/brain imaging
6. Make the diagnosis of posterior reversible encephalopathy syndrome (PRES)
7. Begin appropriate blood pressure management with IV antihypertensives
8. Obtain a neurology consultation
9. Admit the patient to the intensive care unit (ICU)

**Case Title:** Posterior reversible encephalopathy syndrome (PRES)

**Chief Complaint:** A 60-year-old female who presents with a headache

**General Appearance:** 60-year-old woman in a dark room with her eyes closed in mild distress

### Primary Survey

- **Airway:** Patent
- **Breathing:** Clear to auscultation bilaterally, no increased work of breathing
- **Circulation:** 2+ radial pulses bilaterally, brisk cap refill in all extremities, mild tachycardia
- **Disability:** No focal neurologic deficit, alert and oriented

### History

- **History of present illness**: The patient is a 60-year-old woman with a complaint of a headache. The patient states that she was at home watching television when she began to have a headache. The patient specifically states that she does not usually get headaches. Her headache was slow in onset, bilateral without radiation and began earlier today, several hours ago. The headache is a 6/10 on a pain scale. It is different from prior headaches and associated with photophobia, phonophobia and blurry vision. She denies any weakness or sensation changes. There are no alleviating or aggravating factors.
- **Past medical history:** Hypertension, diabetes mellitus type 2, end stage renal disease (ESRD)
- **Past surgical history:** Renal transplant 4 years ago
- **Patient’s medications:** Metformin 500 mg twice daily, Amlodipine 5mg daily, Clonidine 0.1mg daily, Cyclosporine 250 mg twice daily
- **Allergies:** No known drug allergies
- **Social history:** Lives with husband. Never a smoker. Denies any alcohol or illicit drug use.
- **Family history:** Father with hypertension and diabetes. Mother with glaucoma.

### Secondary Survey/Physical Examination

- **General appearance:** appears stated age; laying on hospital bed in a dark room with her eyes closed in mild distress
- **Head, ears, eyes, nose and throat (HEENT):**
  ○ **Head:** atraumatic, normocephalic
  ○ **Eyes:** pupils equal (5mm) and reactive to light, external ocular movements are normal, normal conjunctiva, no papilledema, intraocular pressure 15mmHg bilaterally
  ○ **Ears:** within normal limits
  ○ **Nose:** within normal limits
  ○ **Throat:** no erythema or edema of the oropharynx
- **Neck:** supple, no masses, midline trachea, full range of motion, no jugular venous distention
- **Heart:** regular rhythm with mild tachycardia, no audible murmurs
- **Lungs:** clear to auscultation bilaterally, no increased work of breathing
- **Abdominal/GI:** soft, nontender, not distended, no guarding or rigidity
- **Genitourinary:** within normal limits
- **Rectal:** within normal limits
- **Extremities:** no lower extremity edema, negative Homan’s sign, no lower extremity edema, no cyanosis
- **Back:** nontender, no step-offs, normal range of motion
- **Neuro:** alert and oriented x4, cranial nerves II-XII grossly intact, extra-ocular movements intact, Vision 20/20 bilaterally, 5/5 strength in all extremities, sensation intact to light touch in all extremities, able to walk without difficulty, Glasgow Coma Scale (GCS) 15
- **Skin:** within normal limits
- **Lymph:** within normal limits
- **Psych:** within normal limits

### Results

*Electrocardiogram (ECG)* (author’s own image)

*Chest X-ray:* Normal (author’s own image)

*CT Head wo Contrast-Axial* (author’s own image)

*MRI Brain T2 FLAIR-Axial* (author’s own image)

*MRI Brain T2 Flair-Coronal* (author’s own image)

## OPERATOR MATERIALS

### Diagnosis

Posterior reversible encephalopathy syndrome (PRES), grand mal seizure, refractory headache, hypertensive emergency

### Disposition

Admit to the intensive care unit (ICU)

## DEBRIEFING AND EVALUATION PEARLS

### Posterior Reversible Encephalopathy Syndrome (PRES)

**Pathophysiology:** Three leading hypotheses exist but the pathophysiology remains controversial.

1. Autoregulation Failure: As the upper limit of cerebral autoregulation is exceeded, cerebral blood flow increases with increases in systolic blood pressure (SBP). This cerebral hyper-perfusion causes breakdown of the blood brain barrier.
2. Cerebral Ischemia: The disordered cerebral autoregulation results in local focal vasoconstriction which leads to local ischemia and cytotoxic edema.
3. Endothelial Dysfunction: Underlying cause of PRES leads to toxicity of vascular endothelium and subsequent vasogenic edema and blood brain barrier dysfunction.

#### Risk Factors

- Hypertension: Hypertension associated with autoregulatory failure and is thought to be a key factor in the development of PRES. It is thought that acute changes in blood pressure are related to PRES.
- Immunosuppressive Therapy: Approximately 50% of PRES patients are on immunosuppressant therapy; most cases are associated with cyclosporine use.
- Renal Disease: PRES is often associated with end-stage renal disease and is most associated with lupus nephritis and glomerulonephritis.

**Clinical Features**: PRES has been noted in patients aged 2 to 90 years, but is most commonly described in young to middle-aged females. Clinical features vary significantly but most often start with neurological symptoms.

- Headache: Constant, nonlocalized, and frequently refractory to analgesics and other typical migraine abortive agents
- Altered Consciousness: Ranging from somnolence to agitation, or coma
- Visual Disturbances: Can include blurred vision, hemianopia, visual hallucinations, auras, cortical blindness, or others
- Seizures: Generalized tonic-clonic seizures can recur and can progress to status epilepticus

**Laboratory Studies**: No particular laboratory study can diagnose PRES; however, laboratory studies are often obtained to evaluate for other causes of altered mental status, headache, or seizures

**Radiographic Findings**: Radiographic imaging is the gold-standard of diagnosis

- CT Scan: Classically, white matter edema is present in the posterior cerebral hemispheres often with cerebellar or brainstem involvement. Importantly, the white matter edema is not localized to a single vascular territory. It is not uncommon for a CT scan without contrast to be read as “normal.”
  MRI: Classically, shows focal areas of increased signal on T1 weighted MRI with leptomeningeal signal enhancement, which is thought to be indicative of blood-brain barrier disruption. Fluid-attenuated inversion recovery (FLAIR) sequences show focal but usually symmetric hemisphere vasogenic edema involving white matter. The severity of presentation has not been shown to correlate with the severity of edema seen on MRI.

#### Treatment

Hypertension: Goal to lower diastolic blood pressure to 100–105 mmHg within 2–6 hours with initial fall in BP not exceeding 25%. Most effectively achieved with IV anti-hypertensive agents. Recommended agents are titratable IV drips.

1. Nicardipine: Calcium channel blocker
  - Advantages: Good for intracranial activity
  - Disadvantages: Slower onset and longer half-life
  - Dosage: start 5mg/hour IV, titrating up 2.5mg/hour every 15 minutes up to 15mg/hr
2. Labetalol: Beta/alpha blocker
  - Advantages: Does not significantly affect heart rate or cerebral flow, rapid onset
  - Disadvantages: Should be avoided in asthma/COPD or hyperadrenergic states (ie, pheochromocytoma, cocaine use)
  - Dosage: 0.5–2mg/minute IV
3. Nitroprusside: Vasodilator
  - Advantages: Very fast onset and offset, very effective
  - Disadvantages: Cyanide toxicity, can increase heart rate. Contraindicated in pregnancy
  - Dosage: 0.3–0.5 mcg/kg/min IV, increase by 0.5mcg/kg/min every 5 minutes. Maximum dose is 10mcg/kg/min for 10 minutes

Seizures: Overall management should mirror other seizure management. Protect the patient from injury. Place him/her in the recovery position. Use supplemental oxygen. Support the airway.

- 1^st^ line – benzodiazepine
- 2^nd^ line – levetiracetam, phenytoin, fosphenytoin, or valproic acid
- 3^rd^ line – propofol, midazolam, ketamine, phenobarbital

Headaches: Overall management should mirror the management of a refractory, nonspecific headache.

- 1^st^ line – analgesics, antiemetics (prochlorperazine or metoclopramide) and diphenhydramine
  ○ Analgesics (acetaminophen, NSAIDs), avoiding opiates
  ○ Sumatriptan
  ○ Steroids (dexamethasone, solumedrol)
- 2^nd^ and 3^rd^ line
  ○ Magnesium sulfate
  ○ Valproic acid
  ○ Haloperidol
  ○ Olanzapine
  ○ Ketamine
  ○ Cervical spine injection, sphenopalatine ganglion block, or greater occipital nerve block

Discontinue Immunosuppressive Therapy: Reducing or discontinuing immunosuppressants my help to discontinue seizures, headaches, and help with blood pressure management. Such decisions should not be undertaken without consulting a transplant specialist.

#### Other debriefing points

- Discuss how learners felt regarding communication amongst the participants, especially if there was confusion regarding the diagnosis.
- If the hospital has a protocol for PRES, the instructors should be prepared to discuss this protocol.

## SIMULATION ASSESSMENT

*Posterior Reversible Encephalopathy Syndrome (PRES)*

Learner: _________________________________________

***Assessment Timeline***

This timeline is to help observers assess their learners. It allows observer to make notes on when learners performed various tasks, which can help guide debriefing discussion.

**Critical Actions**:

□ Connect the patient to the cardiac monitor and obtain a full set of initial vital signs
□ Obtain IV access and laboratory studies
□ Symptomatically treat the patient’s headache – antiemetics, analgesics (avoiding opioids), antihistamines, vasodilators, abortive agents, etc.
□ Manage a patient with a seizure – including supplemental oxygen, recovery position, possibly benzodiazepines or other antiepileptic agents
□ Obtain appropriate head/brain imaging
□ Make the diagnosis of posterior reversible encephalopathy syndrome (PRES)
□ Begin appropriate blood pressure management with IV antihypertensives
□ Obtain a neurology consultation
□ Admit the patient to the intensive care unit (ICU)

**Summative and formative comments:**

**Milestones assessment**:
