# Supplementary material for: Posterior Reversible Encephalopathy Syndrome (PRES)
Source: J Educ Teach Emerg Med. 2020 Jan 15;6(1):S46–73. doi: 10.21980/J85W6C (PMC10332763; doi:10.21980/J85W6C)
Supplement: Supplementary file 1 [file jetem-6-1-s46-supp1.pptx]

## Slide 1
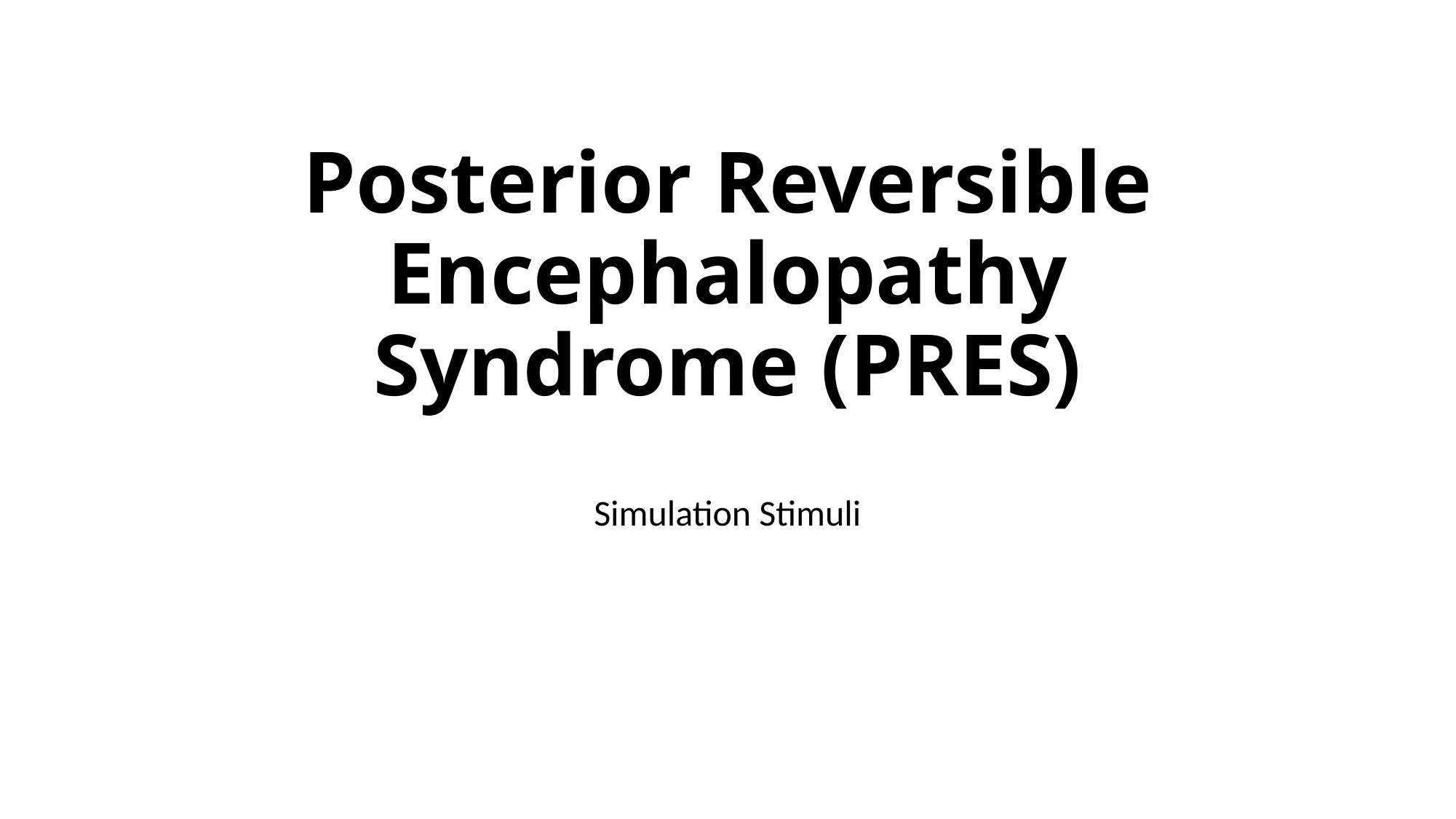

# Posterior Reversible Encephalopathy Syndrome (PRES)
Simulation Stimuli

## Slide 2
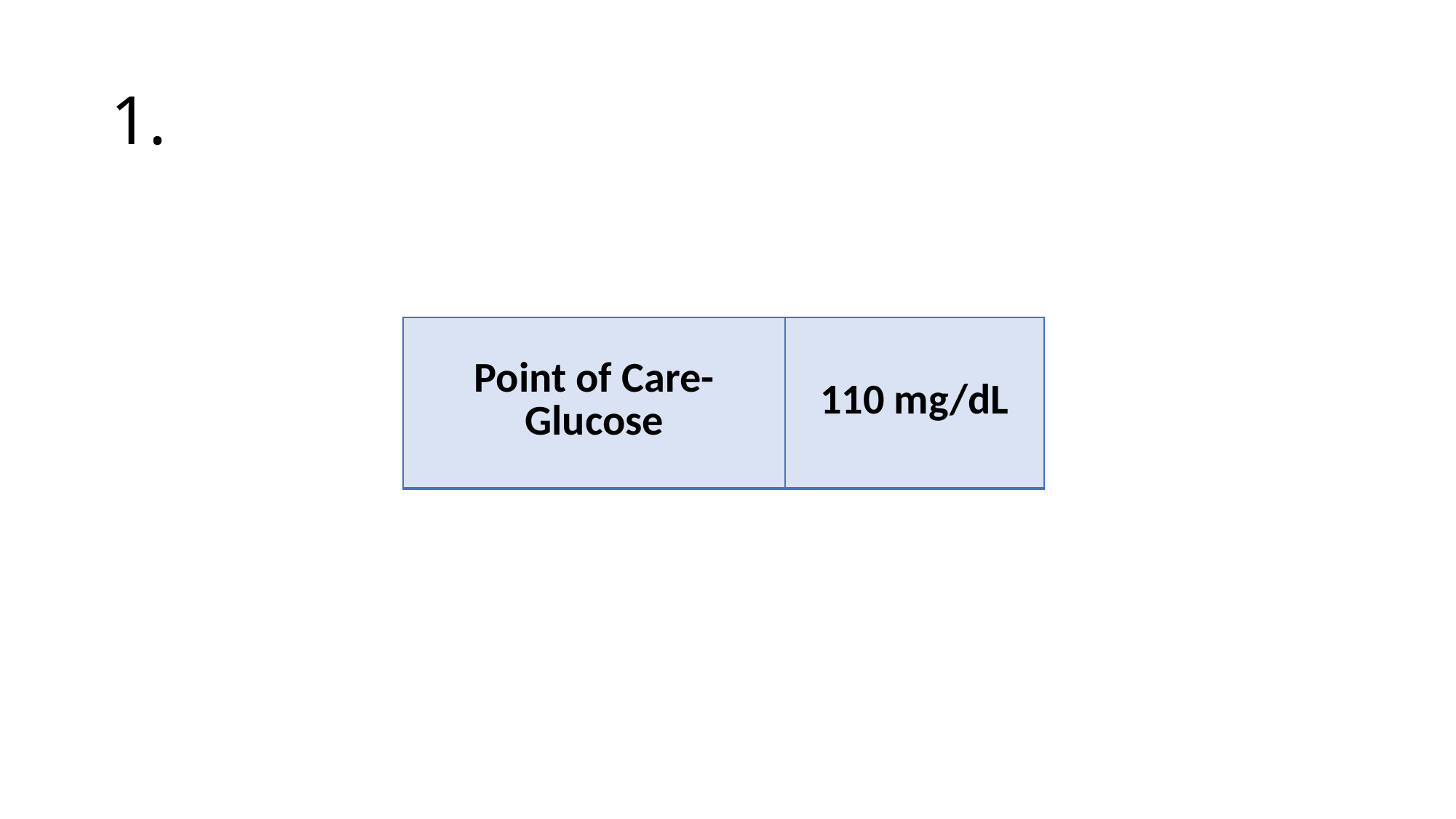

1.
# 1.
| Point of Care- Glucose | 110 mg/dL |
| --- | --- |

## Slide 3
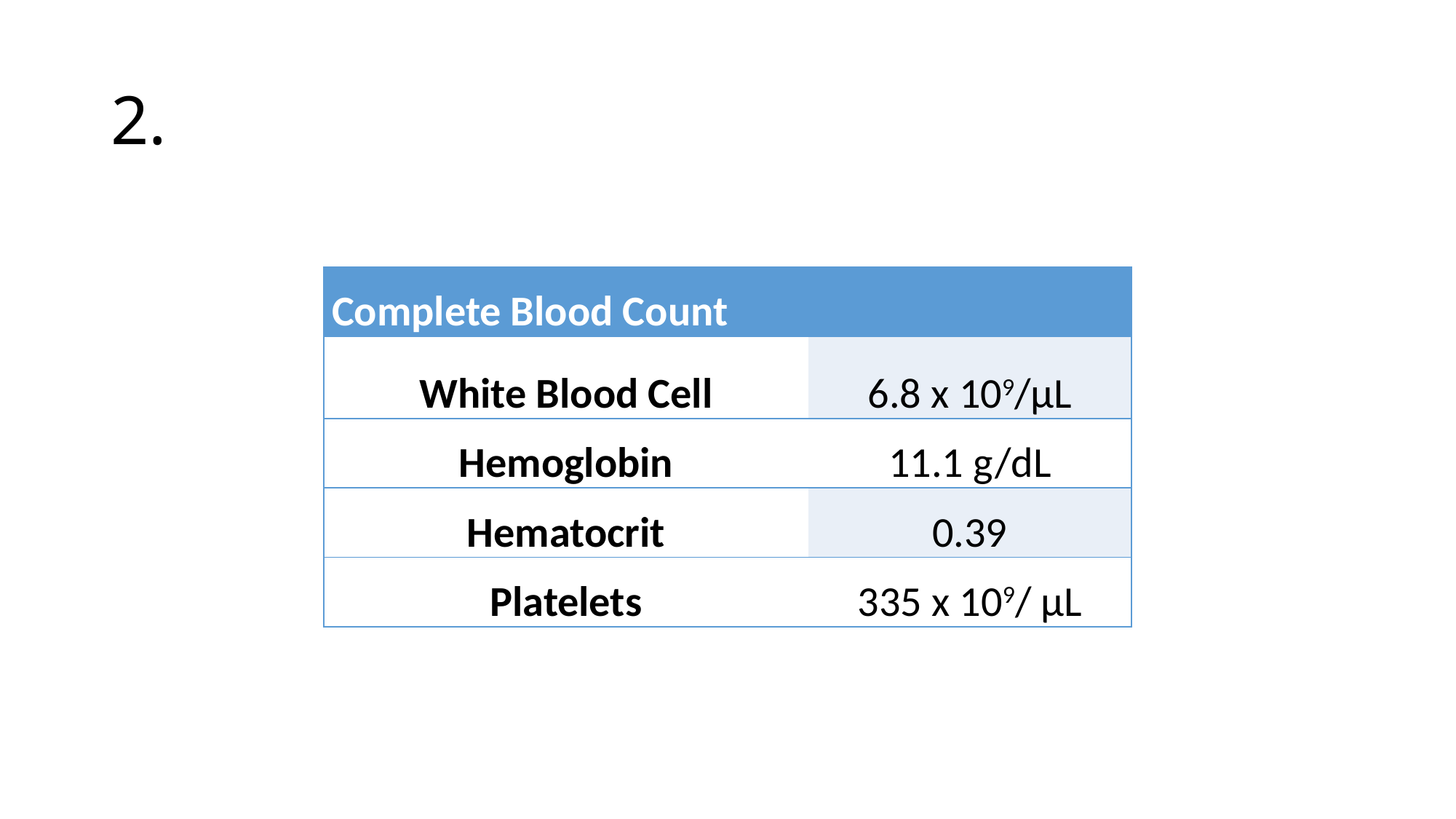

# 2.
| Complete Blood Count | |
| --- | --- |
| White Blood Cell | 6.8 x 10^9^/µL |
| Hemoglobin | 11.1 g/dL |
| Hematocrit | 0.39 |
| Platelets | 335 x 109/ µL |

## Slide 4
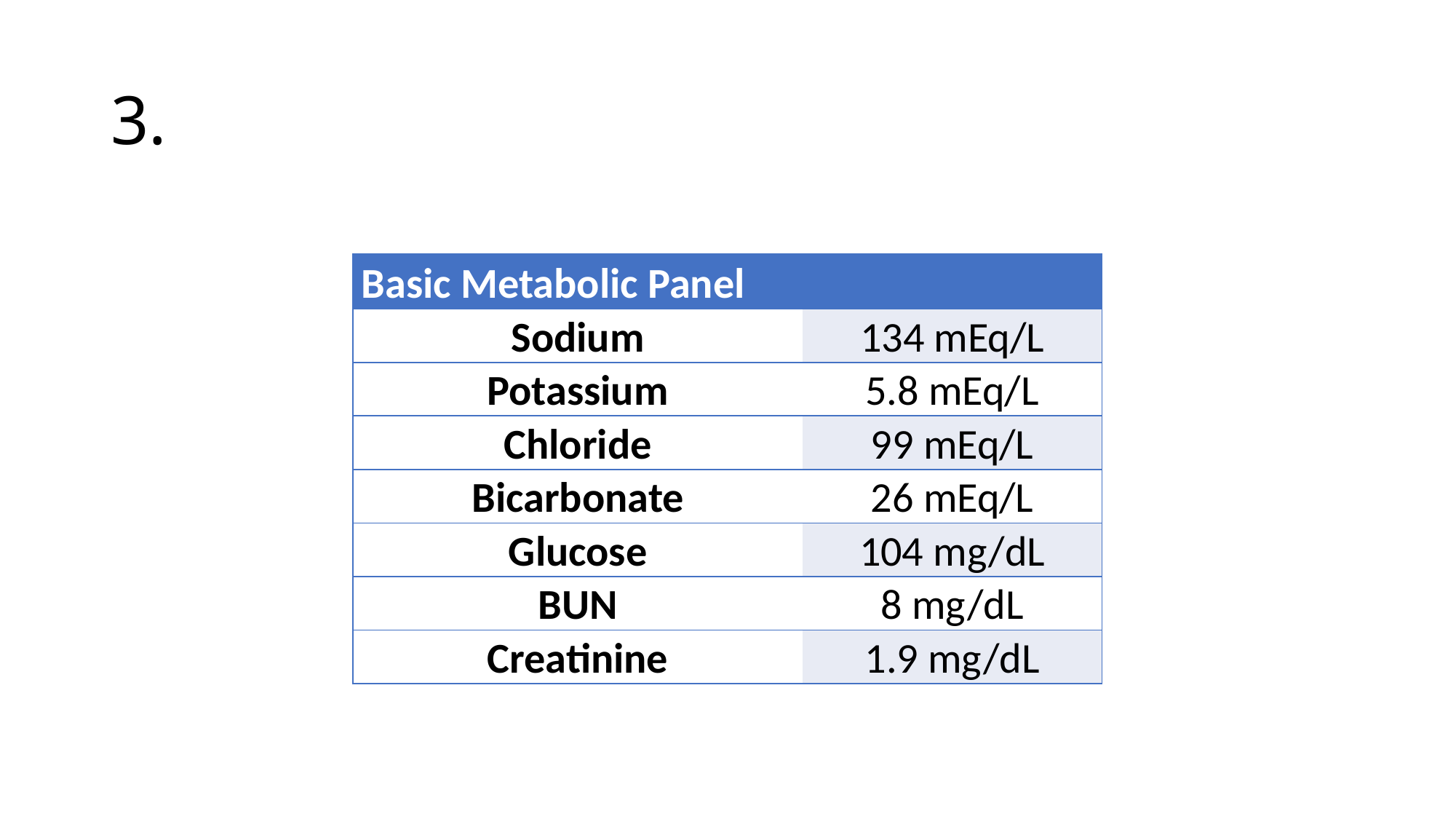

# 3.
| Basic Metabolic Panel | |
| --- | --- |
| Sodium | 134 mEq/L |
| Potassium | 5.8 mEq/L |
| Chloride | 99 mEq/L |
| Bicarbonate | 26 mEq/L |
| Glucose | 104 mg/dL |
| BUN | 8 mg/dL |
| Creatinine | 1.9 mg/dL |

## Slide 5
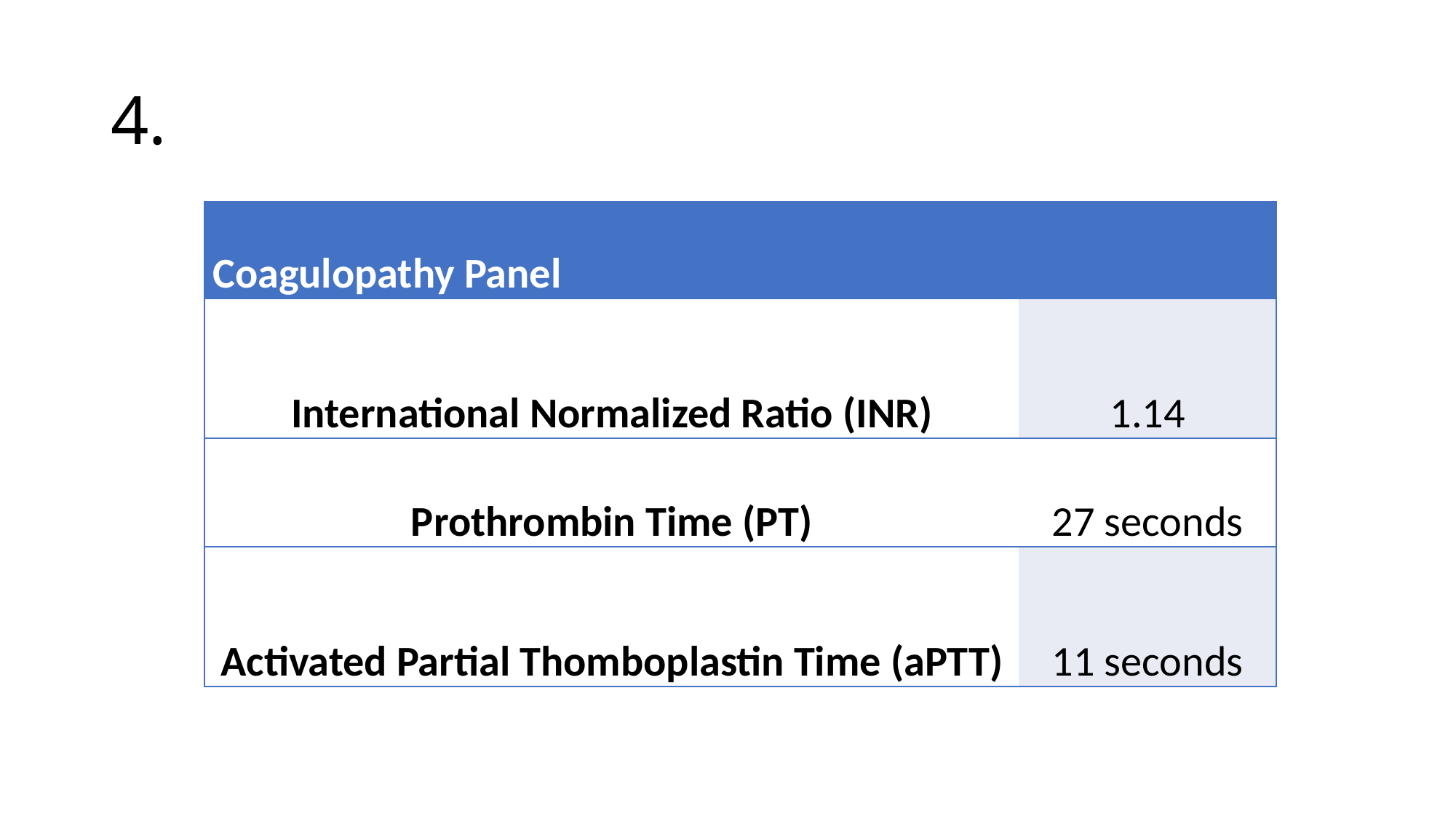

# 4.
| Coagulopathy Panel | |
| --- | --- |
| International Normalized Ratio (INR) | 1.14 |
| Prothrombin Time (PT) | 27 seconds |
| Activated Partial Thomboplastin Time (aPTT) | 11 seconds |

## Slide 6
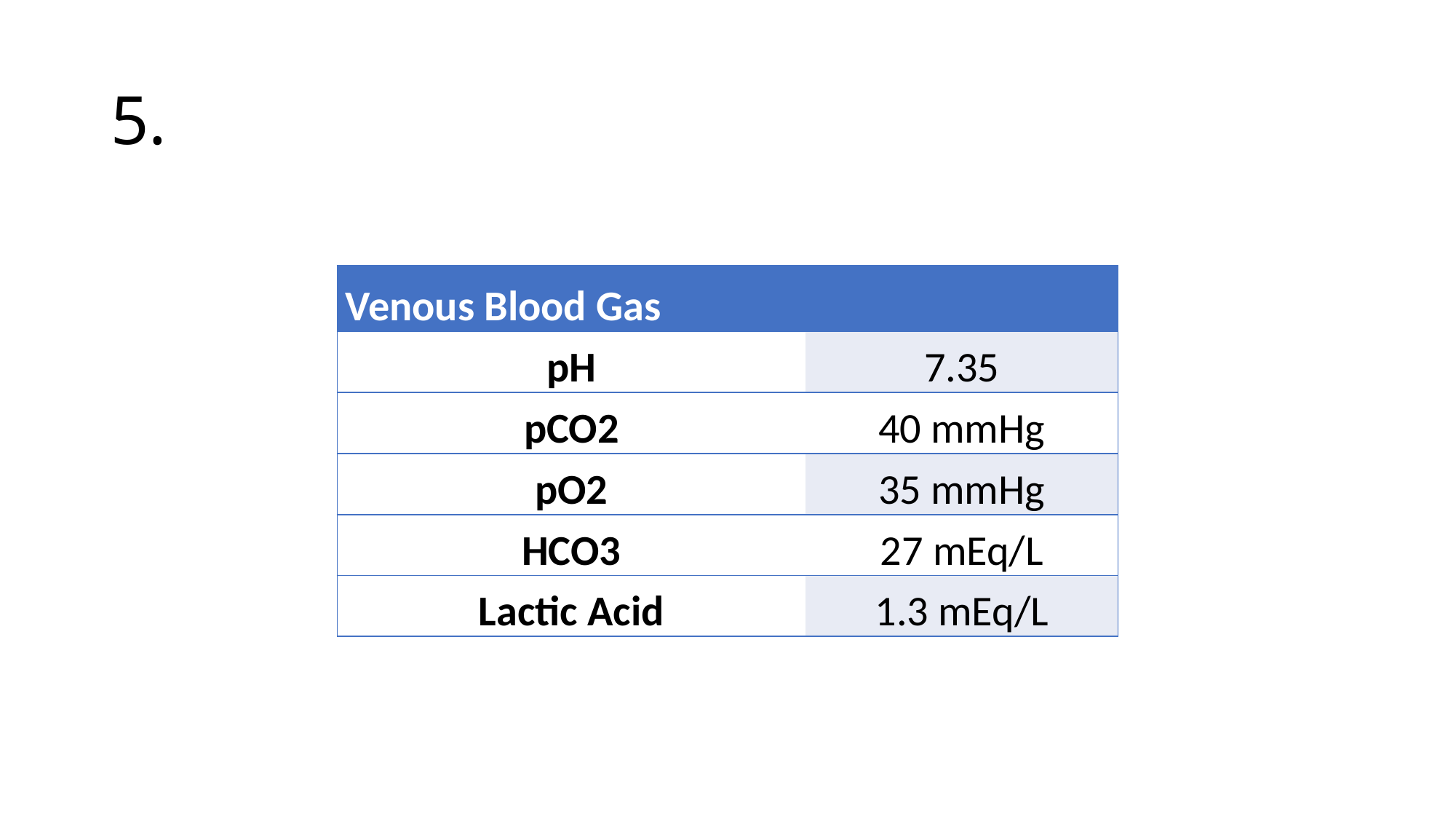

# 5.
| Venous Blood Gas | |
| --- | --- |
| pH | 7.35 |
| pCO2 | 40 mmHg |
| pO2 | 35 mmHg |
| HCO3 | 27 mEq/L |
| Lactic Acid | 1.3 mEq/L |

## Slide 7
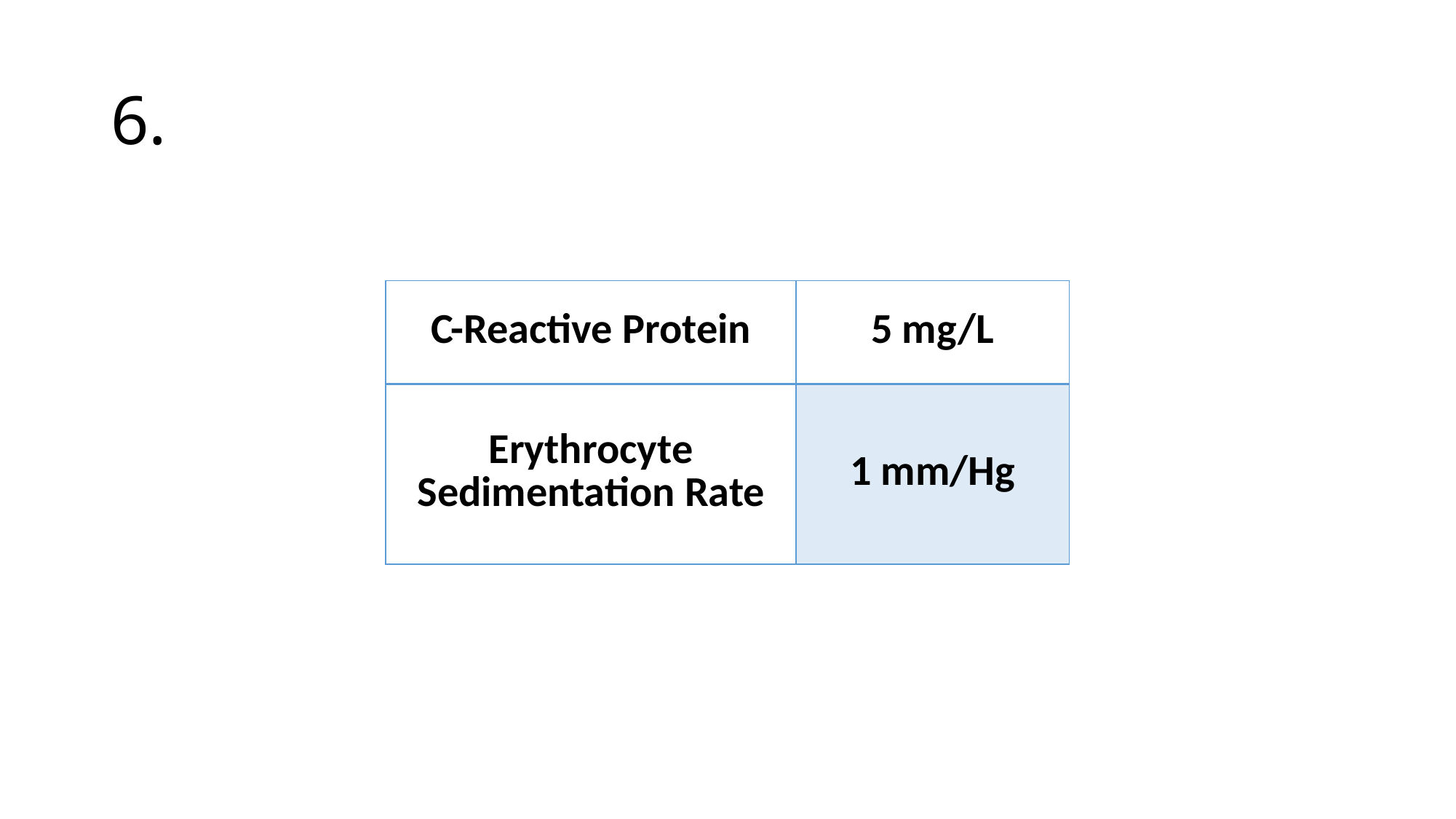

# 6.
| C-Reactive Protein | 5 mg/L |
| --- | --- |
| Erythrocyte Sedimentation Rate | 1 mm/Hg |

## Slide 8
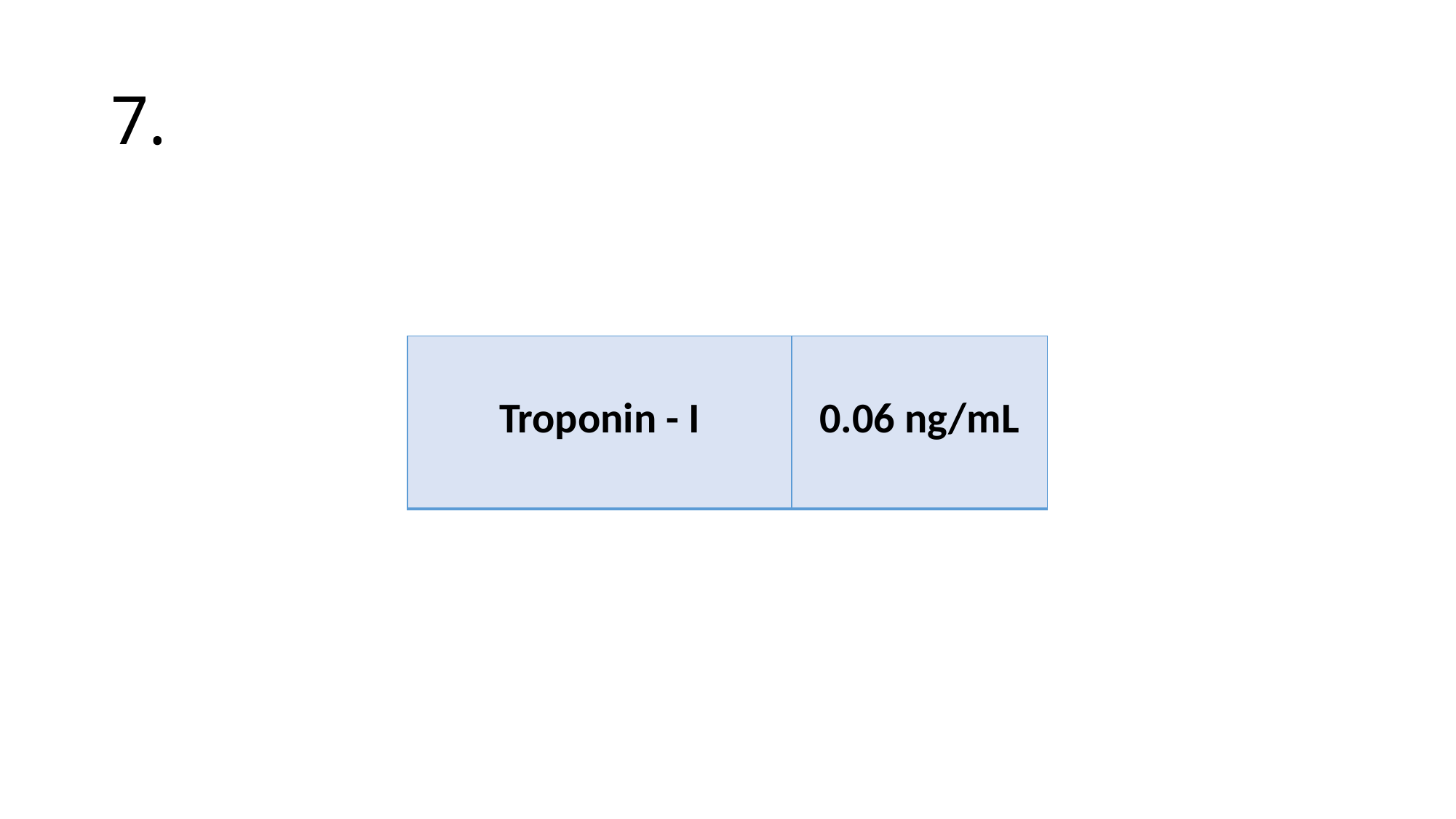

# 7.
| Troponin - I | 0.06 ng/mL |
| --- | --- |

## Slide 9
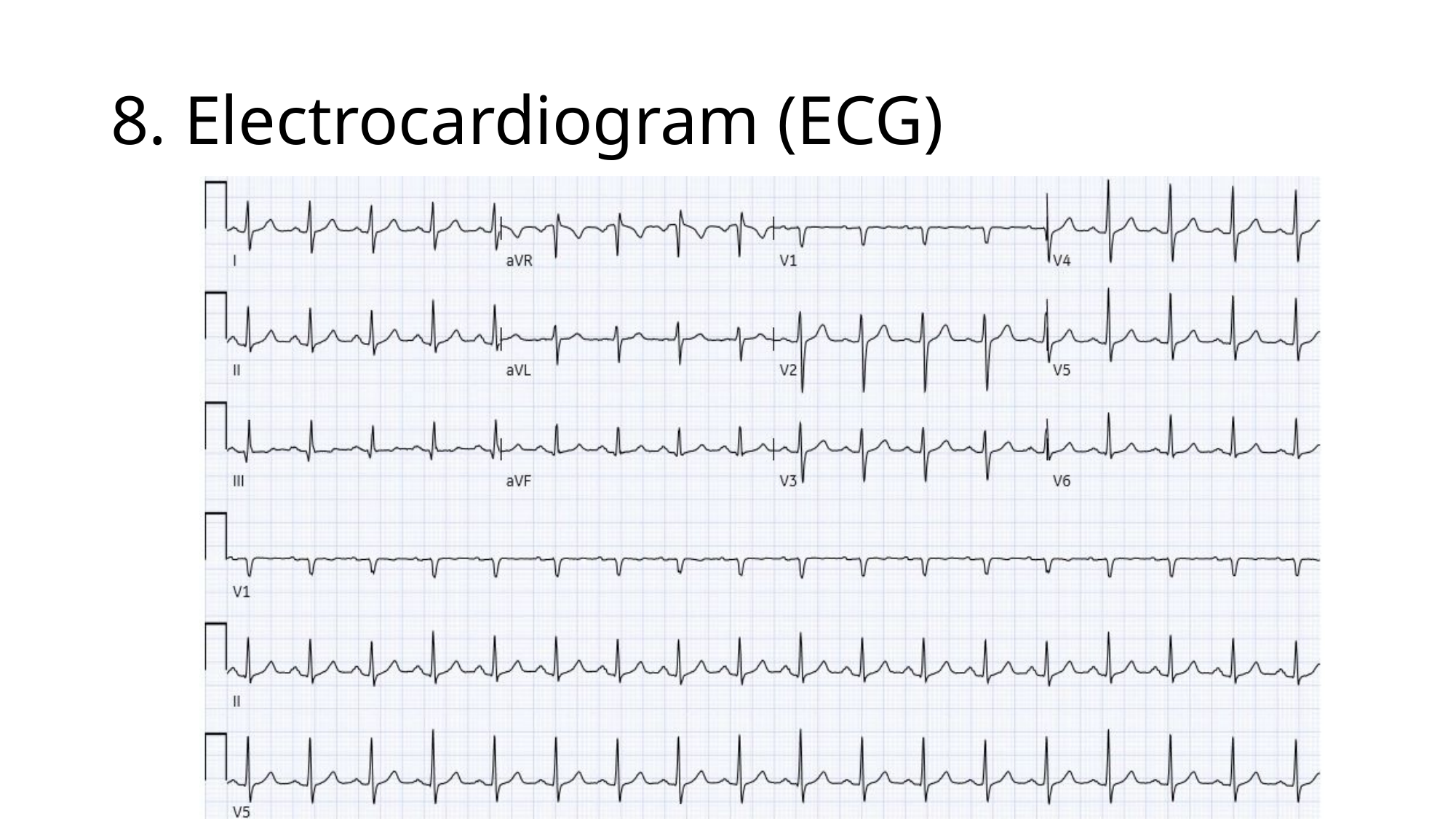

# 8. Electrocardiogram (ECG)

## Slide 10
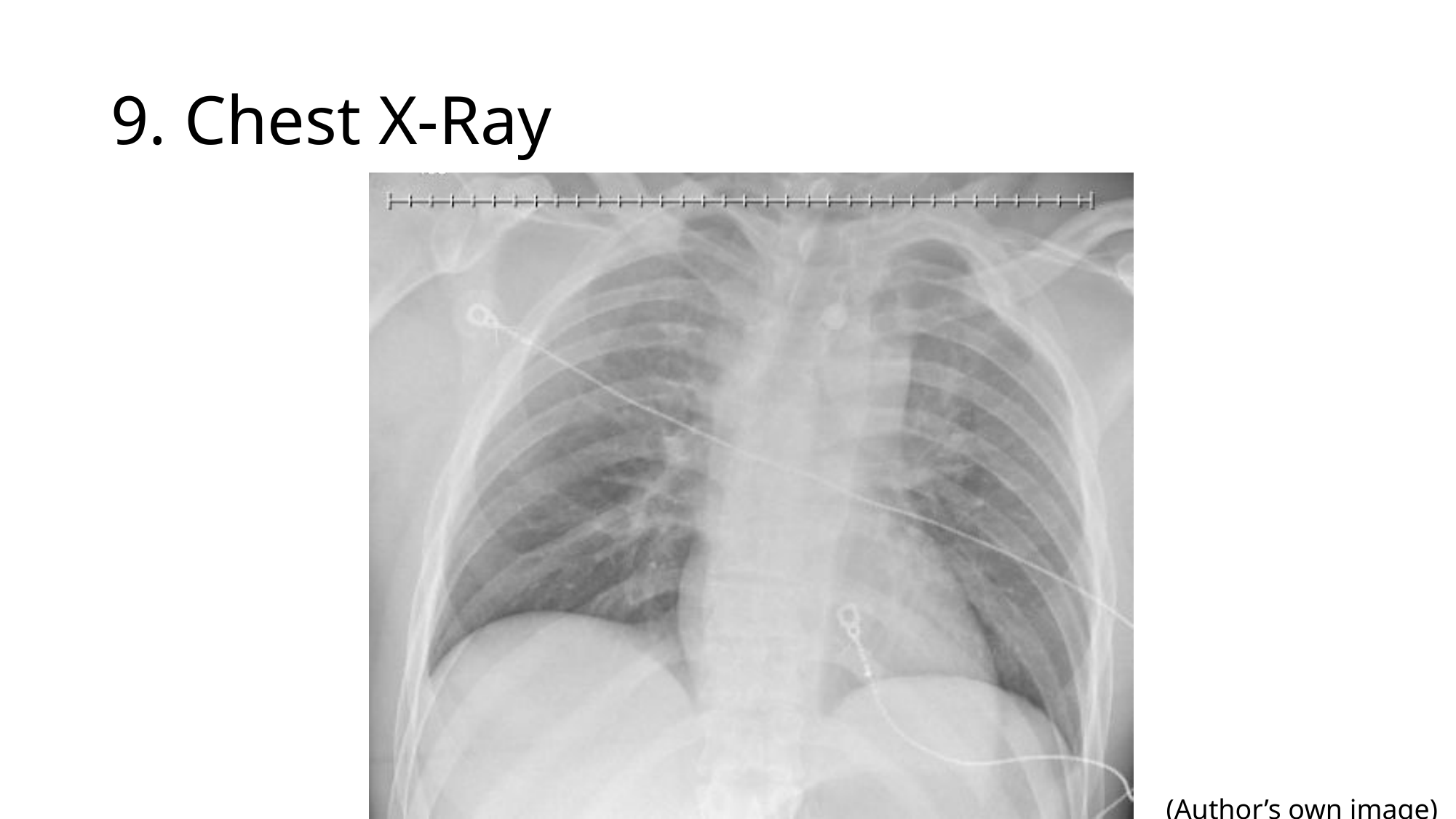

# 9. Chest X-Ray
(Author’s own image)

## Slide 11
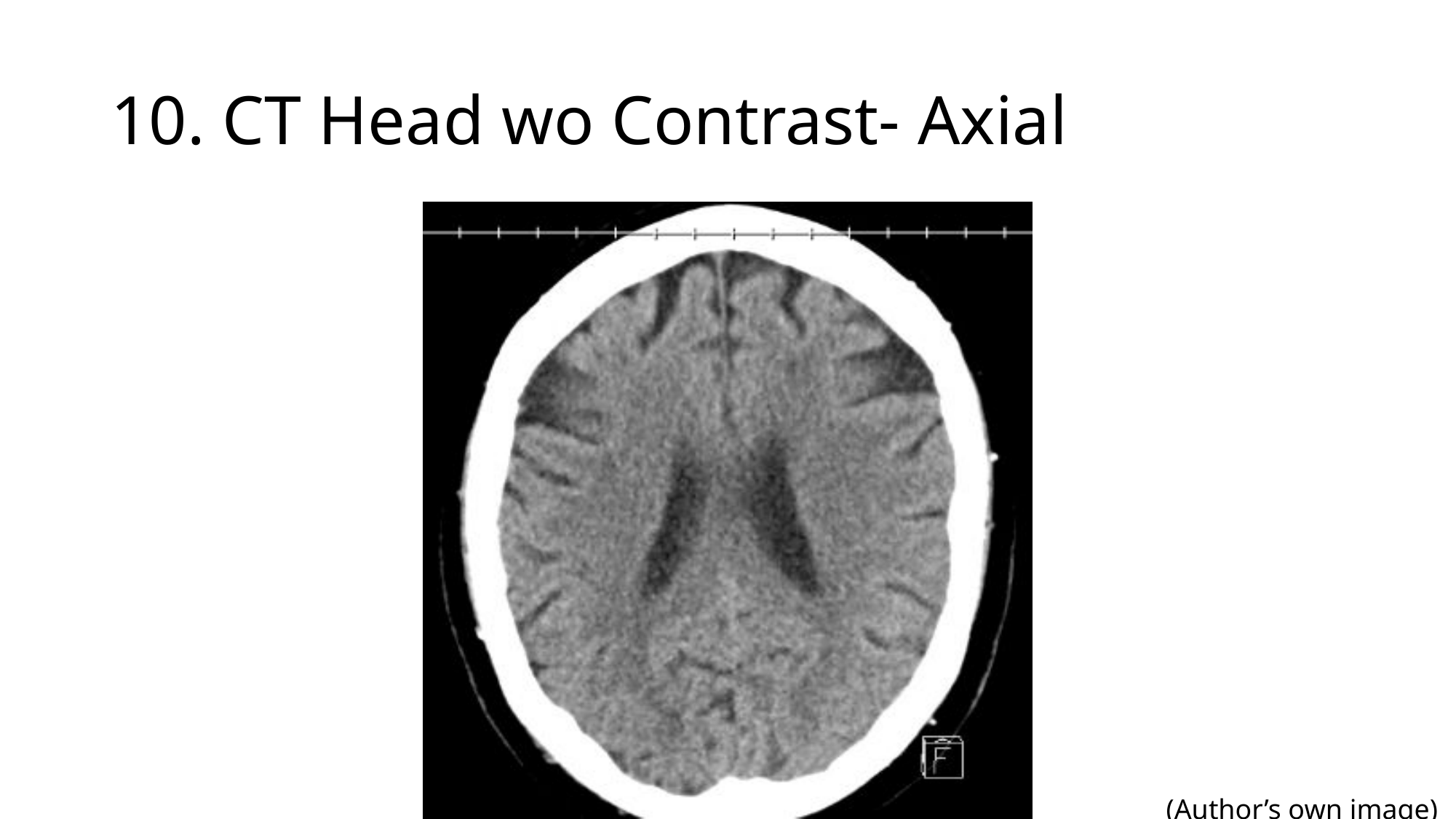

# 10. CT Head wo Contrast- Axial
(Author’s own image)

## Slide 12
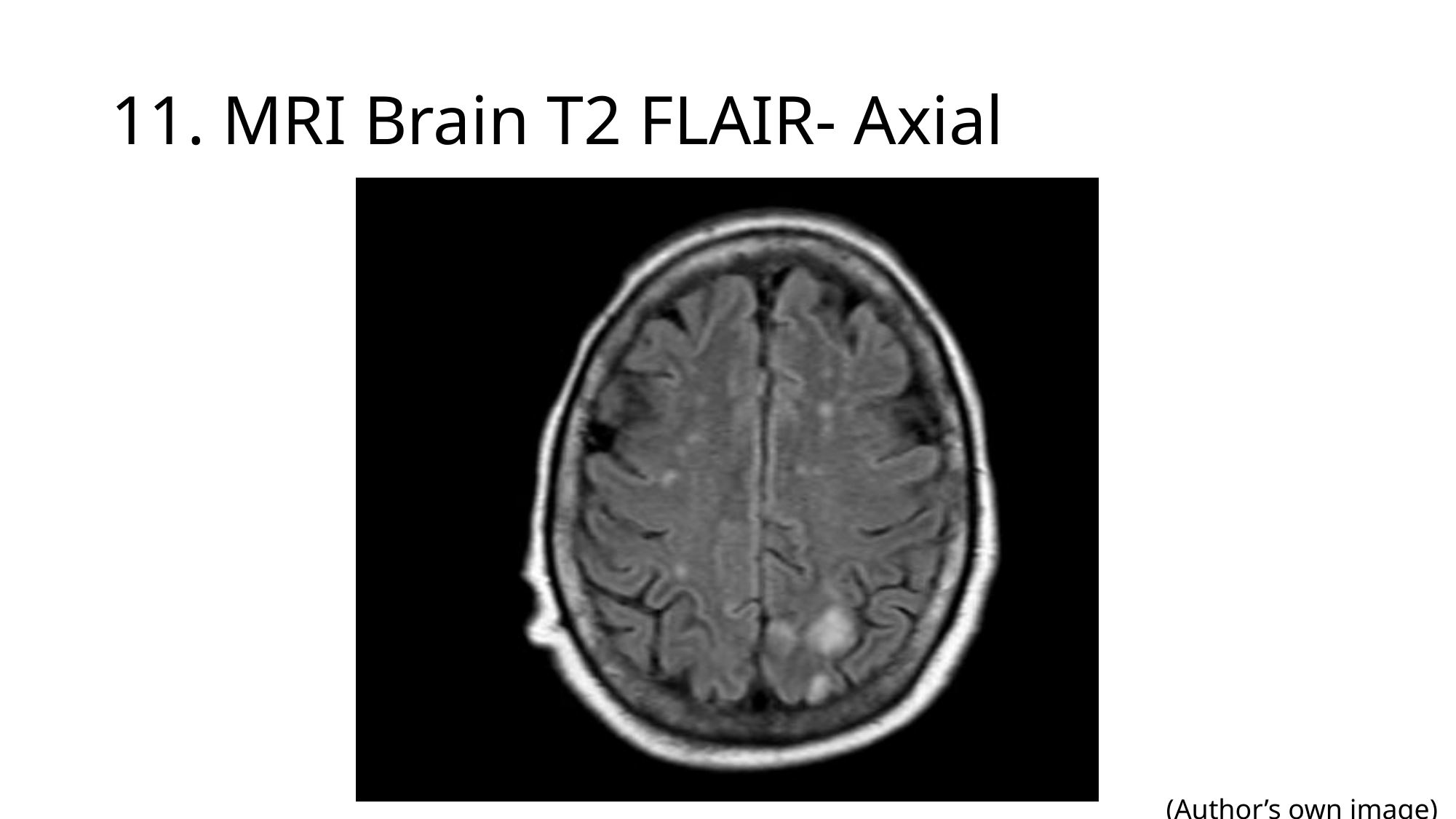

# 11. MRI Brain T2 FLAIR- Axial
(Author’s own image)

## Slide 13
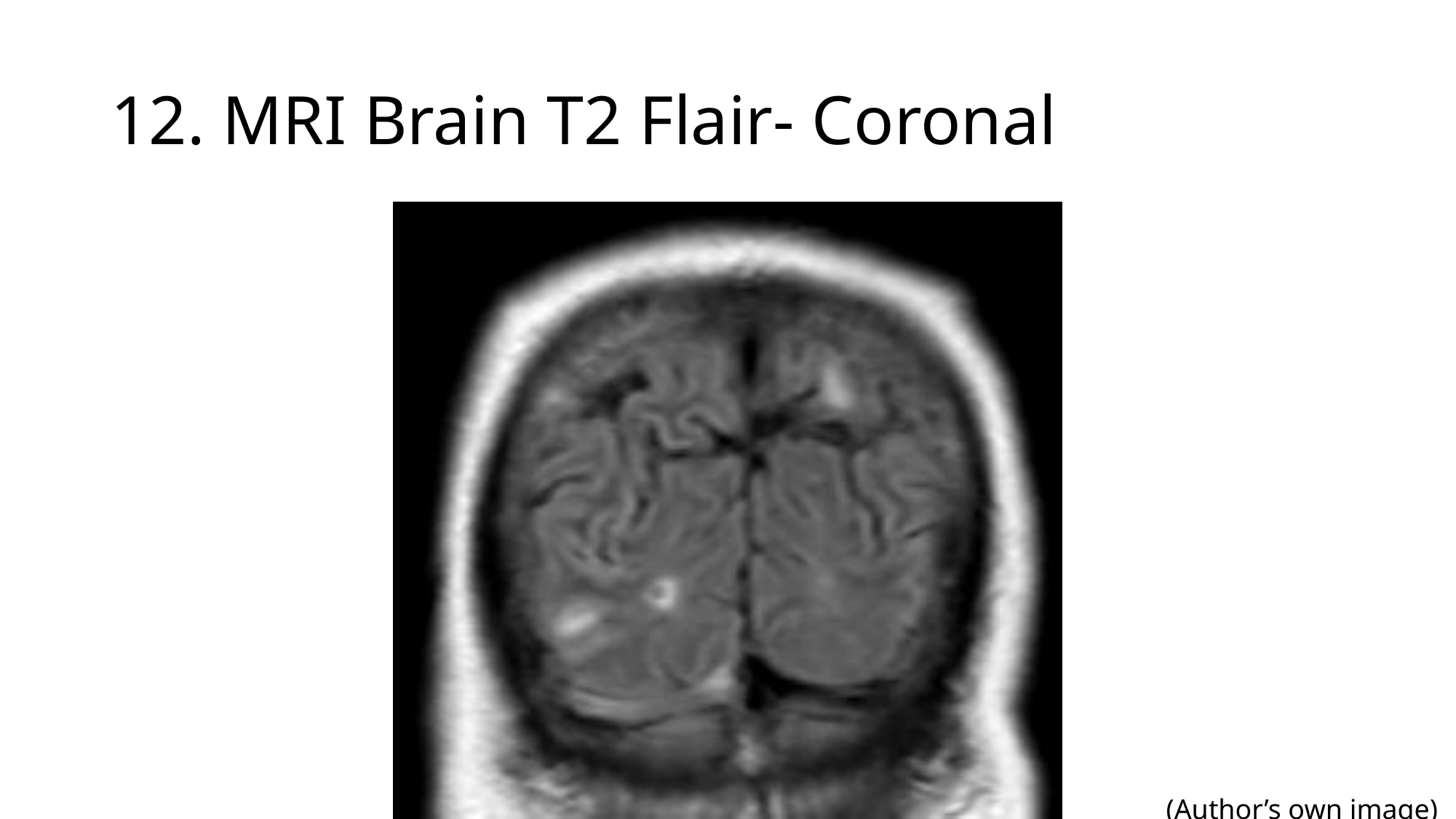

# 12. MRI Brain T2 Flair- Coronal
(Author’s own image)
